# In vitro evaluation of pedicle screw loosening mechanism: a preliminary study on animal model

**DOI:** 10.1186/1748-7161-10-S1-O25

**Published:** 2015-01-19

**Authors:** Hedayeh N Mehmanparast, Jean-Marc Mac-Thiong, Yvan Petit

**Affiliations:** 1 Mechanical Engineering Department, Ecole de Technologie Superieure (ETS), Montreal, Canada; 2 Division of Orthopedic Surgery, University of Montreal, Canada; 3 Research Center, Sacre Coeur Hospital of Montreal, Canada

## Objective

Pedicle screw fixation is a well-established procedure for various spinal disorders. However, pedicle screws failures are still reported. Therefore, there is a need for a better understanding of the pedicle screw failure mechanism. This study investigates the biomechanical stability of pedicle screws on animal vertebrae with a special focus on the screw loosening mechanism.

## Materials and methods

Eighteen vertebrae were harvested from the lumbar section of six porcine spines ranged from L1 to L3. All vertebrae were instrumented on both pedicles using pedicle screws. Quantitative CT scans were used before instrumentation in order to assess the bone density of each vertebra. Cyclic bending (toggling) load of ± 1mm displacement at 3Hz for 5000 cycles were assigned in two directions including craniocaudal (CC) and transverse (TR) toggling. Twelve instrumented pedicles were selected for CC toggling, twelve pedicles for TR toggling and twelve pedicles were not toggled (NT). All toggled and non-toggled screws were then pulled out at a displacement speed of 5mm/min in longitudinal direction. The peak pullout force and stiffness were computed from the load-displacement curves. Analyses of variance (ANOVA) were performed to investigate the effects of toggling methods and vertebral levels on the pullout force and stiffness.

## Results

The results suggest that, regardless to the toggling method used, the pullout force significantly varies between vertebral levels. The highest pullout forces were observed at L1 (1906±225 N for CC, 1917±151 N for TR and 1998±108 N for NT). The lowest pullout forces were detected at L3 (1646±110 N for CC, 1868±120 N for TR and 1875±178 N for NT). Pedicle screw's pullout force and stiffness were significantly affected by toggling method (p=0.001, p<<0.05) and vertebral level (p=0.001, p<<0.05) respectively based on ANOVA. There was a significant difference in stiffness between CC and TR (p=0.02), CC and NT (p<<0.05), and TR and NT pedicle screws (p=0.002).

## Conclusion

The proposed method allowed biomechanical evaluation of the pedicle screw loosening mechanism in-vitro. The pullout force was significantly influenced by vertebral level. Toggling method is more likely to affect pedicle screw stiffness than pullout force. However, conducing further experimental tests are needed to confirm these findings.

